# Transient Global Ventricular Hypertrophy in a Patient With Multisystem Inflammatory Syndrome in Children (MIS-C) Correlated With High-Dose Glucocorticoid Treatment: A Case Report

**DOI:** 10.7759/cureus.32139

**Published:** 2022-12-02

**Authors:** Zachary E West, Matthew Dove, Lazaros K Kochilas, Matthew E Oster

**Affiliations:** 1 Department of Pediatrics, Emory University School of Medicine, Atlanta, USA; 2 Division of Pediatric Cardiology, Children's Healthcare of Atlanta, Atlanta, USA

**Keywords:** covid 19, mis-c, multisystem inflammatory syndrome in children, covid, transient ventricular hypertrophy, glucocorticoids

## Abstract

Multisystem inflammatory syndrome in children (MIS-C) following SARS-CoV-2 infection has been shown to lead to depressed cardiac function. Standard treatment includes high-dose glucocorticoids (GC). We present the unusual case of a teenager who developed transient echocardiographic global ventricular hypertrophy following GC administration during his treatment for MIS-C, with the resolution of the hypertrophy after cessation of GC.

## Introduction

Multisystem inflammatory syndrome in children (MIS-C) following SARS-CoV-2 infection can have severe cardiac involvement, including myocarditis, pericarditis, arrhythmias, and coronary artery dilation. According to the MIS-C guidelines put forth by the American College of Rheumatology, intravenous immunoglobulin (IVIG) is the first-line treatment, with the addition of intravenous methylprednisolone if the patient has signs of shock or organ-threatening disease [1]. Glucocorticoids (GC) have been shown to be an effective and safe treatment for MIS-C [2]. Case reports [3-5] and studies [6-13] have identified ventricular and/or septal hypertrophy after GC treatment in neonates treated for bronchopulmonary dysplasia (BPD) or for stem cell transplant [14]. A strong link has been found between methylprednisolone and very low birth weight infants developing cardiac hypertrophy while being treated for BPD [6]. In these patients, cardiac hypertrophy is also commonly identified after multiple doses of dexamethasone [7,8]. Yet, there have been no published cases of this phenomenon in association with treatment for MIS-C. We present a rare case of transient cardiac hypertrophy in a teenager after MIS-C therapy, including glucocorticoids.

## Case presentation

A 17-year-old male with a positive SARS-CoV-2 reverse transcriptase polymerase chain reaction (PCR) test 46 days prior to admission presented with a six-day history of fever with a maximum temperature of 104⁰F and a three-day history of non-bloody, non-bilious emesis, diarrhea, and abdominal pain. He was brought to the emergency department (ED) after developing poor oral intake and diminished urine output. Upon arrival to the ED, he was hypotensive (78/52 mm Hg), tachycardic (133 bpm), and minimally responsive to two liters of isotonic fluid administration at a rate of 2000 mL/hr. An abdominal CT showed diffuse bowel wall thickening and borderline cardiomegaly. He was transferred to the pediatric intensive care unit at a tertiary care children’s hospital. Laboratory evaluation showed elevated inflammatory markers (C-reactive protein 15.2 mg/dl (normal <1.0 mg/dl), procalcitonin 18 ng/ml (normal <0.10 ng/ml)), as well as markers of myocardial damage and ischemia (troponin 37.3 ng/mL (normal <0.045 ng/mL) and B-natriuretic peptide 691 pg/ml (normal <100 pg/ml)). SARS-CoV-2 PCR was negative, but SARS-CoV-2 anti-nucleocapsid IgG was positive. With multiorgan involvement, laboratory evidence of inflammation, recent COVID-19 illness, and no other plausible diagnosis, he met the criteria for the diagnosis of MIS-C per CDC guidelines.

**Figure 1:**
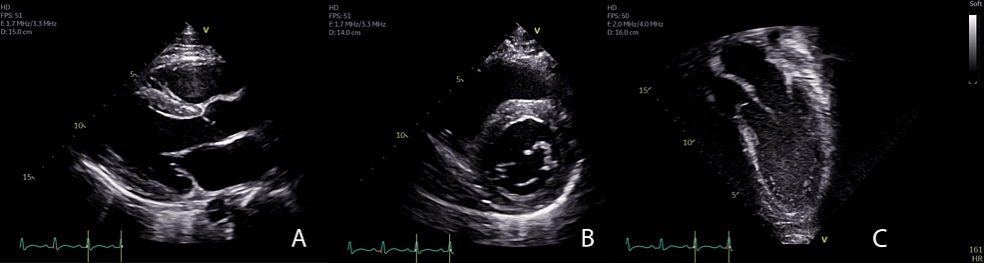
Echocardiogram at initial presentation Transthoracic echocardiography of the parasternal long axis (A), parasternal short axis (B), and apical three chamber view (C) were obtained on day one of hospitalization prior to initiation of treatment for MIS-C. There was no hypertrophy of the left ventricle.

As per hospital protocol for evaluation and treatment of MIS-C, he underwent a transthoracic echocardiogram (TTE) on the day of admission, which showed a structurally normal heart with normal coronary arteries, moderately depressed biventricular systolic performance (left ventricular (LV) ejection fraction 40% by Simpson’s biplane method), mild mitral regurgitation, normal biventricular size, and normal interventricular septal and posterior wall thickness (interventricular septum thickness in diastole (IVSd): 1.13 cm, z score 1.24; LV posterior wall thickness in diastole (LVPWd): 1.07 cm, z score 1.45) (Figure 1, Video 1).

**Video 1:** Echocardiogram at initial presentation Transthoracic echocardiography of the parasternal long axis was obtained on day one of hospitalization prior to initiation of treatment for MIS-C. There was no hypertrophy of the left ventricle.

**Figure 2:**
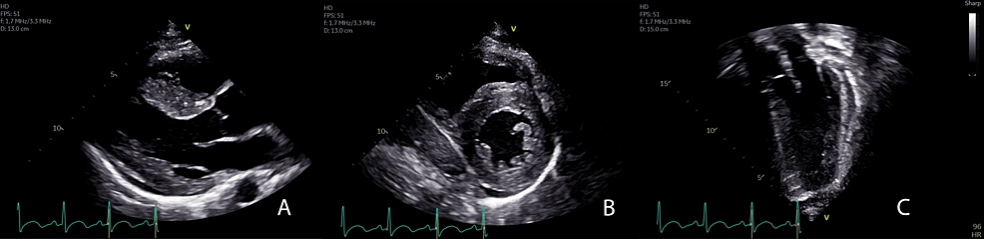
Echocardiogram demonstrating global left ventricular hypertrophy Transthoracic echocardiography of the parasternal long axis (A), parasternal short axis (B), and apical three-chamber view (C) were obtained on day eight of hospitalization while undergoing treatment for MIS-C. There was global hypertrophy of the left ventricle.

The electrocardiogram showed sinus tachycardia at 130 bpm with non-specific T-wave abnormalities and no evidence of right or left ventricular hypertrophy. The patient was treated with continuous epinephrine and milrinone infusions to manage hypotension and cardiac dysfunction. He was given an intravenous immunoglobulin (IVIG) infusion of 100 g and IV methylprednisolone 60 mg every 12 hours to treat the hyperinflammation associated with MIS-C. TTEs performed on hospital day two and five were similar to the TTE on admission. On hospital day eight, TTE showed normalization of LV systolic function, but there was new global ventricular hypertrophy with moderate basal septal hypertrophy (IVSd 1.53 cm; z score 4.05) without evidence of dynamic right or left ventricular outflow tract obstruction (Figure 2, Video 2).

**Video 2:** Echocardiogram demonstrating global left ventricular hypertrophy Transthoracic echocardiography of the parasternal long axis was obtained on day eight of hospitalization while undergoing treatment for MIS-C. There was asymmetric hypertrophy of the left ventricle.

**Figure 3:**
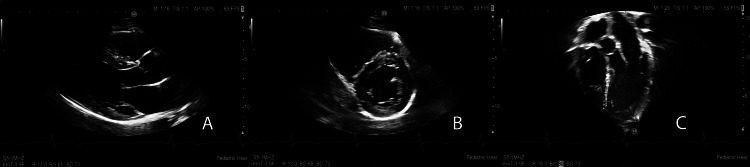
Echocardiogram after resolution of ventricular hypertrophy Transthoracic echocardiography of the parasternal long axis (A), parasternal short axis (B), and apical three-chamber view (C) were obtained 45 days after the initial presentation for MIS-C. There was no hypertrophy of the left ventricle.

Inotropic infusions were weaned off after normalization of function. He received a total of eight days of methylprednisolone, followed by a nine-day wean of prednisone. TTE on hospital day 10 revealed the same results as on hospital day eight, and the patient was discharged home. In outpatient cardiology follow-up nine days after discharge, TTE showed normal function and resolution of the ventricular hypertrophy (IVSd 1.18 cm; z = +1.71). At a follow-up visit one month later, the interventricular wall thickness continued to decrease (IVSd 1.02 cm; z score 0.47; Figure 3). An extended ECG rhythm patch was placed at that time due to complaints of palpitations and revealed two episodes of non-sustained ventricular tachycardia, with the longest event lasting nine beats at an average heart rate of 109 bpm. A cardiac MRI was performed four months after diagnosis and revealed normal biventricular size and function. There was no evidence of edema, hyperemia, or myocardial fibrosis. Cardiopulmonary exercise testing performed on the same day as the MRI was normal. He has not been noted to have hypertension.

## Discussion

We present the first reported case of transient global ventricular hypertrophy in association with treatment for MIS-C in a teenage male. The hypertrophy resolved after the completion of a course of treatment with GC without evidence of residual abnormalities.

GC has been associated with inducing cardiac hypertrophy via complex transcriptional activation and physiologic effects and with a notable sex bias for male subjects for such effects [15]. In neonates, septal and ventricular hypertrophy secondary to GC is usually benign and spontaneously resolves two to five weeks after GC is discontinued, though it did resolve earlier in our case [9]. In one study of 31 preterm infants, 94% developed left ventricular hypertrophy, with median hypertrophy of 56% increase in left ventricular posterior wall thickness in diastole (LVPWd), appearing within three days, peaking at ten days, and resolving within 27 days [9]. Left ventricular outflow tract obstruction was not seen in any of these patients [9].

The left ventricular and septal hypertrophy in our patient was improving by the time of his outpatient echocardiogram one week after discharge and had resolved completely one month later. A cardiac MRI several months later showed no ventricular hypertrophy and no lasting effects of MIS-C, consistent with other long-term studies of these patients [16]. Additionally, similar to findings in the majority of case reports in the neonatal population, our patient did not develop left ventricular outflow tract obstruction (LVOTO).

Other cases of hypertrophy with myocarditis have been found to be related to the degree of inflammation leading to edema rather than the use of GC. There have been reports of asymmetric hypertrophy in COVID-19 myocarditis [17] and global ventricular hypertrophy from edema in other forms of myocarditis [18-20]. In fulminant myocarditis, septal hypertrophy has been seen as well [18].

**Figure 4:**
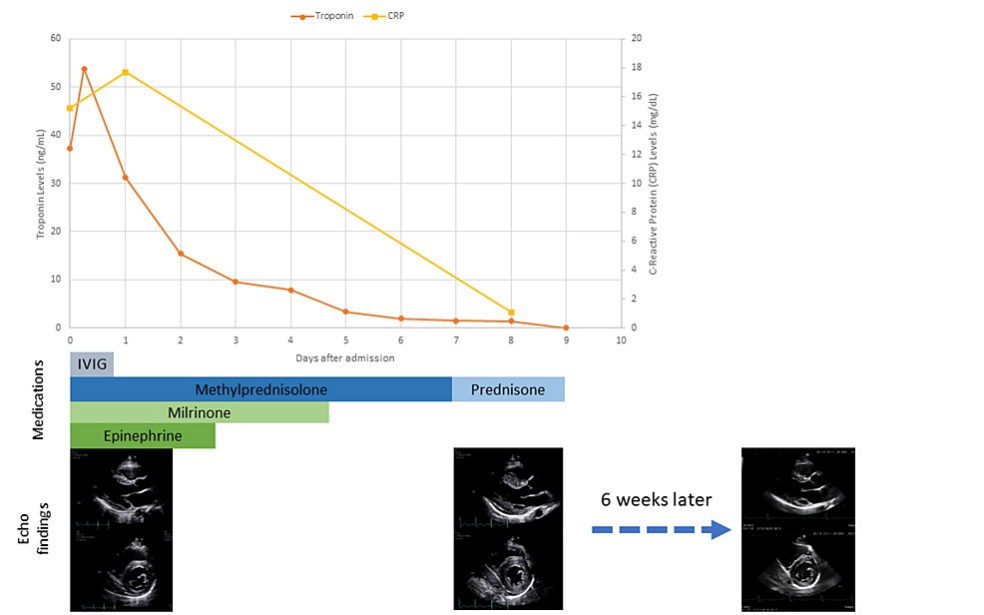
Summary of the hospital course with lab values, medications, and echocardiography images C-reactive protein (CRP), troponin, medications including intravenous immunoglobulin (IVIG), glucocorticoids (methylprednisolone and prednisone), milrinone, and epinephrine, as well as echo findings by the day after admission. Echocardiography images are the parasternal long axis (top) and parasternal short axis (bottom) views from Figures 1, 2, and 3.

Without a cardiac MRI performed while the patient had transient global hypertrophy, it is difficult to discern whether the changes are a result of hypertrophy from steroids or edema from inflammation. Yet, at the time of the development of ventricular hypertrophy in this patient, the inflammatory markers and troponin levels were decreasing, and he had already received several days of high-dose GC (Figure 4). He no longer required vasopressors and was otherwise clinically improving. He also did not have underlying hypertension that could lead to hypertrophy.

## Conclusions

While our patient developed transient global ventricular hypertrophy, it is reassuring that the patient did not develop left ventricular outflow tract obstruction (LVOTO). Our patient did not have symptoms directly related to his transient ventricular hypertrophy, but this etiology should be considered in patients receiving GC. This is especially important in the decision-making process surrounding critical care management, as steps should be taken to maintain afterload given the risk of cardiovascular collapse due to LVOTO if it were to develop. Additionally, with this global hypertrophy, there is a concern for decreased ventricular filling that could lead to a reduced cardiac output due to the reduction in preload. In our case, the patient was on vasopressors for multiple days, which was likely in large part due to the inflammatory process of the MIS-C, but it is worth considering the role of the decreased preload in the need for vasopressors.

The causality of GC as the inciting agent for the observed transient hypertrophy in our case cannot be proven. Nevertheless, the course of events in our patient, along with the described effects of GC on cardiomyocytes and other clinical and experimental settings with GC signaling dysregulation, suggest that the possibility of GC-induced transient ventricular hypertrophy needs to be considered among the differential diagnoses in such patients.

## References

[1] Henderson LA, Canna SW, Friedman KG (2021). American College of Rheumatology clinical guidance for multisystem inflammatory syndrome in children associated with SARS-CoV-2 and hyperinflammation in pediatric COVID-19: version 2. Arthritis Rheumatol.

[2] Villacis-Nunez DS, Jones K, Jabbar A (2022). Short-term outcomes of corticosteroid monotherapy in multisystem inflammatory syndrome in children. JAMA Pediatr.

[3] Gill AW, Warner G, Bull L (1996). Iatrogenic neonatal hypertrophic cardiomyopathy. Pediatr Cardiol.

[4] Jiang J, Zhang J, Kang M, Yang J (2019). Transient hypertrophic cardiomyopathy and hypertension associated with hydrocortisone in preterm infant: a case report. Medicine (Baltimore).

[5] Kale Y, Aydemir O, Ceylan O, Bas AY, Demirel N (2015). Hypertrophic cardiomyopathy after a single dose of dexamethasone in a preterm infant. Pediatr Neonatol.

[6] Dani C, Bertini G, Simone P, Rubaltelli FF (2006). Hypertrophic cardiomyopathy in preterm infants treated with methylprednisolone for bronchopulmonary dysplasia. Pediatrics.

[7] Halliday HL, Ehrenkranz RA, Doyle LW (2003). Early postnatal (<96 hours) corticosteroids for preventing chronic lung disease in preterm infants. Cochrane Database Syst Rev.

[8] Halliday HL, Ehrenkranz RA, Doyle LW (2003). Moderately early (7-14 days) postnatal corticosteroids for preventing chronic lung disease in preterm infants. Cochrane Database Syst Rev.

[9] Skelton R, Gill AB, Parsons JM (1998). Cardiac effects of short course dexamethasone in preterm infants. Arch Dis Child Fetal Neonatal Ed.

[10] Zecca E, Papacci P, Maggio L, Gallini F, Elia S, De Rosa G, Romagnoli C (2001). Cardiac adverse effects of early dexamethasone treatment in preterm infants: a randomized clinical trial. J Clin Pharmacol.

[11] Bloomfield FH, Knight DB, Harding JE (1998). Side effects of 2 different dexamethasone courses for preterm infants at risk of chronic lung disease: a randomized trial. J Pediatr.

[12] Werner JC, Sicard RE, Hansen TW, Solomon E, Cowett RM, Oh W (1992). Hypertrophic cardiomyopathy associated with dexamethasone therapy for bronchopulmonary dysplasia. J Pediatr.

[13] Miranda-Mallea J, Pérez-Verdú J, Gascó-Lacalle B, Sáez-Palacios JM, Fernández-Gilino C, Izquierdo-Macián I (1997). Hypertrophic cardiomyopathy in preterm infants treated with dexamethasone. Eur J Pediatr.

[14] Lesnik JJ, Singh GK, Balfour IC, Wall DA (2001). Steroid-induced hypertrophic cardiomyopathy following stem cell transplantation in a neonate: a case report. Bone Marrow Transplant.

[15] Yang J, Chen Y, Li X, Xu D (2022). New insights into the roles of glucocorticoid signaling dysregulation in pathological cardiac hypertrophy. Heart Fail Rev.

[16] Capone CA, Misra N, Ganigara M (2021). Six month follow-up of patients with multi-system inflammatory syndrome in children. Pediatrics.

[17] Edwards JJ, Harris MA, Toib A, Burstein DS, Rossano JW (2022). Asymmetric septal edema masking as hypertrophy in an infant with COVID-19 myocarditis. Prog Pediatr Cardiol.

[18] Felker GM, Boehmer JP, Hruban RH, Hutchins GM, Kasper EK, Baughman KL, Hare JM (2000). Echocardiographic findings in fulminant and acute myocarditis. J Am Coll Cardiol.

[19] Pinamonti B, Alberti E, Cigalotto A, Dreas L, Salvi A, Silvestri F, Camerini F (1988). Echocardiographic findings in myocarditis. Am J Cardiol.

[20] Morimoto S, Kato S, Hiramitsu S (2003). Narrowing of the left ventricular cavity associated with transient ventricular wall thickening reduces stroke volume in patients with acute myocarditis. Circ J.

